# Engineered biomaterials in stem cell-based regenerative medicine

**DOI:** 10.1093/lifemedi/lnad027

**Published:** 2023-07-20

**Authors:** Fei Zhu, Guangjun Nie, Changsheng Liu

**Affiliations:** CAS Key Laboratory for Biomedical Effects of Nanomaterials & Nanosafety, CAS Centre for Excellence in Nanoscience, National Centre for Nanoscience and Technology, Beijing 100190, China; CAS Key Laboratory for Biomedical Effects of Nanomaterials & Nanosafety, CAS Centre for Excellence in Nanoscience, National Centre for Nanoscience and Technology, Beijing 100190, China; Center of Materials Science and Optoelectronics Engineering, University of Chinese Academy of Sciences, Beijing 100049, China; Key Laboratory for Ultrafine Materials of Ministry of Education, East China University of Science and Technology, Shanghai 200237, China

**Keywords:** stem cells, engineered biomaterials, stem cell-based regenerative therapy, adjustable biophysical and biochemical properties, regenerative medicine

## Abstract

Stem cell-based regenerative therapies, which harness the self-renewal and differentiation properties of stem cells, have been in the spotlight due to their widespread applications in treating degenerative, aging, and other, generally intractable diseases. Therapeutically effective hematopoietic stem cells, mesenchymal stem cells, embryonic stem cells, and induced pluripotent stem cells have been used in numerous basic and translational studies with exciting results. However, pre-/post-transplantation issues of poor cell survival and retention, uncontrolled differentiation, and insufficient numbers of cells engrafted into host tissues are the major challenges in stem cell-based regenerative therapies. Engineered biomaterials have adjustable biochemical and biophysical properties that significantly affect cell behaviors, such as cell engraftment, survival, migration, and differentiation outcomes, thereby enhancing the engraftment of implanted stem cells and guiding tissue regeneration. Therefore, the combination of stem cell biology with bioengineered materials is a promising strategy to improve the therapeutic outcomes of stem cell-based regenerative therapy. In this review, we summarize the advances in the modulation of behaviors of stem cells via engineered biomaterials. We then present different approaches to harnessing bioengineered materials to enhance the transplantation of stem cells. Finally, we will provide future directions in regenerative therapy using stem cells.

## Introduction

**Figure 1.**
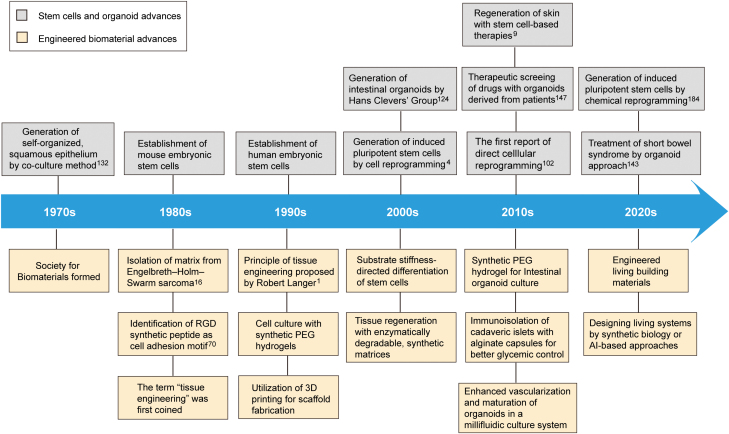
Timeline for the major advances in engineered biomaterials, stem cells or stem cell-derived organoids.

Regenerative medicine integrates a wide range of research areas, such as stem cells, developmental biology, synthetic biology, materials science, and tissue engineering [1, 2] (Fig. 1). In the past decades, there has been increasing evidence that stem cell-based treatments are an effective approach to regenerative medicine [2, 3]. Stem cells, such as embryonic stem cells (ESCs) [4, 5], induced pluripotent stem cells (iPSCs) [6–8], and adult stem cells (ASCs) (mesenchymal stem cells [MSCs] [9], hematopoietic stem cells [HSCs] [10], and neural stem cells [NSCs] [11]), have the unique capabilities to self-renewal and generate specialized offspring during development as well as throughout the life of an organism. Rapid advancements in the stem cell field in the last 30 years have led to the discovery of unique ways to form cells of interest and even organoids in the lab using patient-derived cell samples [3]. Paradigm for the stem cell research has dramatically changed with the advent of iPSC technology, by which adult cells could be desirably reprogrammed into iPSCs by expressing a set of pluripotency-associated transcription factors [6–8]. The emergence of cell reprogramming techniques has allowed research scientists to obtain individual-specific iPSCs from diversified cells of an individual’s own samples [3]. iPSCs have the capability to, in principle, differentiate into diversified cell types, thereby paving the way for the ultimate objective of individualized, regenerative treatment. Moreover, individual person-derived iPSCs or directly reprogrammed cells may be utilized to functionally screen for therapeutic drugs [3].

The pioneering research of transforming stem cell-based treatment into patient applications has raised expectations that stem cell therapy will heal degenerative, aging, and even some refractory diseases in the future [12] (Table 1). HSCs are clinically transplanted to cure patients with myeloma, lymphoma, leukemia, or autoimmune diseases [13]. Hirsch and colleagues prepared autologous transgenic epidermal stem cells for repairing the majority of damaged epidermis in a patient being diagnosed with a devastating, life-threatening form of junctional epidermolysis bullosa [14]. Differentiation of ESCs or individual person-derived iPSCs into retinal pigmented epithelium, which was then transplanted into the eye, thereby improving vision in blind patients due to neovascular age-related macular degeneration [15, 16].

**Table 1.** Representative stem cell-based regenerative therapies approved or in clinical trials for treating damaged, aging, or intractable diseases

Cell resources	Conditions or diseases	Clinical trial ID	First posted	Clinical stage
iPSC-derived retinal pigmented epithelial cells	Age-related macular degeneration	NCT02464956	8 June 2015	Completed
iPSC-derived retinal pigmented epithelial cells	Age-related macular degeneration	NCT04339764	9 April 2020	Recruiting
iPSC-derived engineered heart myocardium	Heart failure	NCT04396899	21 May 2020	Recruiting
Allogenic human pluripotent stem cell-derived cardiomyocytes	Heart failure	NCT03763136	4 December 2018	Recruiting
Mesenchymoangioblast-derived mesenchymal stem cells	Graft verse host disease	NCT02923375	4 October 2016	Completed
iPSC-derived cancer antigen-specific T cells	Gastrointestinal cancers Breast cancers Pancreatic cancers Melanoma Lung cancers	NCT03407040	23 January 2018	Terminated
HB1.F3-CD neural stem cells	High-grade glioma	NCT02192359	16 July 2014	Active, not recruiting
Human ESC-derived neural precursor cells	Parkinson’s diseases	NCT03119636	18 April 2017	Recruiting
Human neural stem cells	Parkinson’s diseases	NCT02452723	25 May 2015	Active, not recruiting
Human ESC-derived retinal pigmented epithelial cells	Stargardt’s macular dystrophy	NCT01469832	10 November 2011	Completed
Human ESC-derived retinal pigmented epithelial cells	Retinitis pigmentosa	NCT03944239	9 May 2019	Recruiting
Human ESC-derived astrocytes	Amyotrophic lateral sclerosis	NCT03482050	29 March 2018	Completed
Human PSC-derived β cells encapsulated within bioengineered device	Type 1 diabetes mellitus	NCT02239354	12 September 2014	Terminated
Human ESC-derived mesenchymal cells	Intrauterine adhesion	NCT04232592	18 January 2020	Not yet recruiting

Despite the encouraging handful of translational successes of stem cell treatment, widespread application of stem cell treatment has been hindered by several obstacles, including stemness maintenance, large-scale propagation of massive donor cells, robust maintenance of cellular status, and mitigation of undesired inflammatory responses during engraftment, and low surviving of the implanted cells [17]. Bioengineering approaches provide solutions to overcome these obstacles in stem cell-based therapies [17]. Rapid progress in materials science has led to better control of biophysical or biochemical features of engineered biomaterials used for stem cell-based regenerative treatments. The native extra-cellular matrix (ECM) of stem cells has a significant influence on cell survival, propagation, or differentiation [18]. Numerous bioengineered materials have been fabricated to imitate the critical features of native ECM for both basic and translational studies [18]. Because of the capability to imitate the ECM, amicable administration or adjustable features, hydrogels have recently attracted tremendous attention as some of the ideal biomaterials for stem cell regenerative therapies [19].

In this review, the authors summarize the progress of engineered biomaterials for stem cell-based treatment. First, we present the technologies by which bioengineered materials can regulate stem cell fates, followed by the developments in enhancing stem cell transplantation using tailor-made bioengineered materials. Next, we discuss the influence of bioengineered materials on the stem cell derivation of organoids and organoid-based regenerative therapies. Finally, we investigate the challenges and directions of bioengineered material-assisted, stem cell regenerative treatment.

## Massive propagation of stem cells

**Figure 2.**
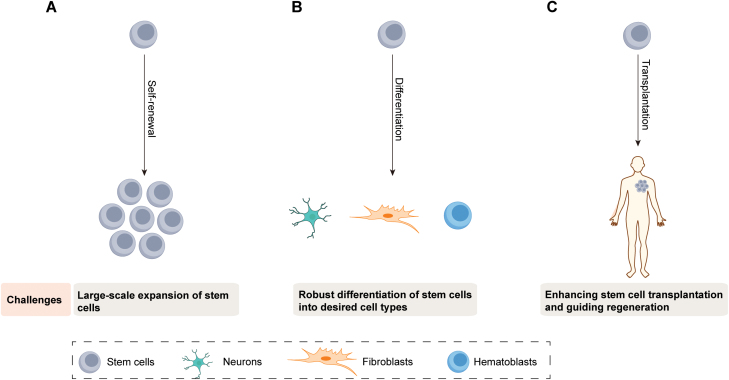
Challenges in translating stem cell therapies. The present challenges facing stem cell therapies include (A) stemness preservation of stem cells and large-scale propagation of stem cells, (B) robust protocols for differentiation of stem cells towards desired cells, and (C) enhancing stem cell transplantation and guiding regeneration.

An important aspect of stem cell regenerative therapies is the massive propagation of the seed cells to gain sufficient quantities of stem cells (Fig. 2A). We need to grow stem cells under fully defined conditions to meet clinical requirements and to minimize lot-to-lot variation to ensure stable efficacy for regenerative therapies [3, 20].

Scalable propagation of pluripotent stem cells (PSCs) of high quality has been considered to be the prerequisite for stem cell-based regenerative therapies [3]. The most widely used method for culturing PSCs contained components of animal origin, including mitotically inactivated cells (or feeder cells) or tissue culture plates coated with Matrigel^TM^, which is extracted from Engelbreth–Holm–Swarm sarcoma and contains ECM ingredients, including laminin, fibronectin, type IV collagen, proteoglycans, and other growth factors [21]. The chemical compositions of these materials are unclear, thereby leading to large batch-to-batch variability and xenogeneic contamination in addition to animal origin [22], which pose great challenges in the utilization of these techniques. Cells cultivated under the aforementioned systems often exhibit abnormal behavior: enlarged morphology, polarization, altered response to drugs, or abnormal karyotype after long-term culture [23]. Cell cultivation systems that offer greater physiological relevance than conventional two-dimensional cultures are needed to close gaps between the current standard cultivation conditions and the native microenvironments [23]. Three-dimensional (3D) cultivation platforms that provide increased space for cell propagation, without leading to unfavorable stacking/aggregation, and favor the large-scale expansion of PSCs are now emerging in the field of engineered biomaterials.

**Figure 3.**
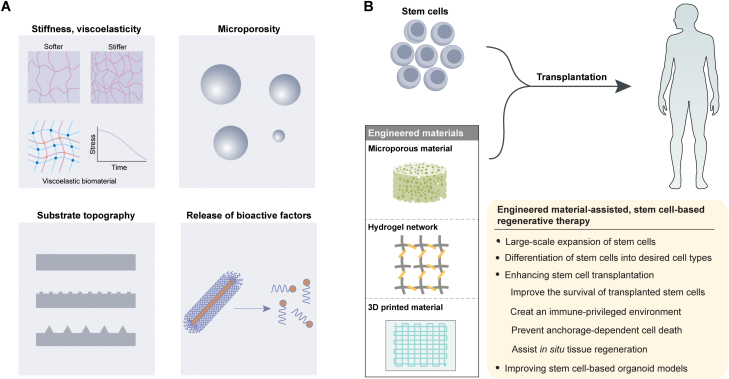
Engineered biomaterials for stem cell-based regenerative therapies. (A) Mechanical characteristics, such as stiffness, viscoelasticity, porous architecture, or topography (nano/micro patterning), can influence a range of cell behaviors, including attachment, expansion, or differentiation of stem cells. Biochemical characteristics, such as the release of bioactive molecules, including growth factors or chemokines, recruit endogenous stem cells to injured sites. (B) Incorporation of engineered materials into stem cell-based regenerative therapies.

The current cultivation system has been significantly improved by the addition of novel engineered biomaterials, which offer different strategies for manipulating biochemical, biophysical, or architectural signals [23] (Fig. 3A), thereby recapitulating the key aspects of *in vivo* milieu [23]. To achieve this goal, different kinds of biomaterials have been fabricated, such as elastic scaffolds, electrospinning materials, bioceramics, patterned substrates, or fiber foams [24]. Hydrogels, referring to a network of the hydrophilic polymer, have been increasingly used for cell cultivation due to their ability to mimic the prominent features of *in vivo* ECM [24, 25]. In one interesting example, Lei and Schaffer reported a chemically defined, expandable, and good manufacturing practice (GMP)-compatible cultivation system for robust propagation of PSCs [26]. Specifically, the authors employed a thermoresponsive hydrogel based on the phase transition behaviour of the poly (N-isopropyl acrylamide)-poly (ethylene glycol, PEG) (PNIPAAm-PEG) hydrogel through transforming temperature between 4°C and 37°C, thereby allowing easy encapsulation and rapid recovery of human PSCs (hPSCs) whenever possible [26]. In similar experiments, massive and low-cost propagation for hPSCs was achieved in a chemically defined, xenogenic-free cultivation platform [20]. This cultivation platform contained well-defined small chemical molecules (1-azakenpaullone, kenpaullone, ID-8, and tacrolimus) and engineered laminin E8 [20]. More recently, Labouesse and colleagues developed a “StemBond” hydrogel system to allow for optimal preservation of PSCs [27]. The authors found that StemBond hydrogels were endowed with adjustable stiffness or tethering of ECM, thereby promoting the strong attachment of PSCs, ultimately facilitating enhanced preservation and prolonged culture of PSCs [27].

Besides biochemical composition, mechanical features of bioengineered materials also have important effects on stem cell behavior [25] (Fig. 3A). One crucial mechanical parameter of bioengineered material is hardness and softness, which describes the ease of deformation of materials under a defined load [25]. The stiffness of biomaterials is described in terms of elastic modulus or Young’s modulus, being defined as the ratio of stress to strain applied at small perturbations [25]. The significant effects of the stiffness of bioengineered materials on modulating stemness preservation and differentiation of PSCs or ASCs have been stringently demonstrated in studies using hydrogels with various stiffness [28–30]. For example, adult muscle stem cells (MuSCs) could retain their optimal phenotype and healing capability when maintained on hydrogels that have comparable elasticity to native muscle tissue [28]. Interestingly, aged MuSCs acquire intrinsic defects in regenerative potential compared with young MuSCs, which hinders progress in addressing the unmet therapeutic need for native MuSCs in elderly individuals [28]. Another study reported the rejuvenation of regenerative capacity in aged MuSCs by coordination of selective inhibition of p38α/β mitogen-activated protein kinase (MAPK) protein within a fabricated scaffold with mechanical properties matching the softness and elasticity of muscle tissue [29]. The authors observed that substrate stiffness in culture had a significant effect on stem cells even after functional engraftment procedures, suggesting stem cells possess a biophysical ‘memory’ [29]. In another noteworthy study, Yang and colleagues synthesized adjustable hydrogels with photodegradable properties based on a poly (ethylene glycol) di-photodegradable acrylate (PEGdiPDA) crosslinker and investigated cell response to changes in the mechanical feature of substrate [30]. The authors found that the influence of the mechanical properties of the substrate on the status of stem cells seems to be coordinated in a cellular autonomous way through yes-associated protein (YAP) signalling [30]. Another elegant study identified the molecular mechanisms that were responsible for this biophysical memory, in which retention of biophysical memory of the past conditions in MSCs was mediated by microRNA (miR-21), the level of which gradually increased during stiff priming [31]. Recently, the effects of substrate degradation and remodeling on stemness preservation or propagation of ASCs have been studied in 3D hydrogel systems that are capable of controllable matrix degradation [32]. The degradation of the substrate was associated with the stemness retention and growth of NSCs [32]. By contrast, stemness retention and proliferation of intestinal stem cells (ISCs) were compromised when cultured in hydrogels susceptible to degradation and matrix remodeling [33].

Another feature of bioengineered materials mimicking *in vivo* ECM is topography, which has been intensively investigated [34–38] (Fig. 3A). ECM of numerous tissues has multiple microscopic structures and exerts important roles in regulating the behavior of stem cells [18]. Research scientists have been working on the fabrication of substrates with different kinds of microscopic structures, such as networks, grooves, columns, pores, or hexagons, to simulate natural microstructure features *in vitro*, thereby investigating the influence of topography on attachment, propagation, migration, or lineage specialization of stem cells [34, 35]. For instance, stemness preservation of cultured MSCs was facilitated by nano-patterned substrates with tetragonal crystal morphologies [36]. Guvendiren and colleagues studied the interplay of MSCs with homogeneous hydrogels or hydrogels with stratiform or hexagonal surface puckers to dissect the influence of topographical properties of hydrogel on the behavior of human MSCs [37]. The authors found that the hydrogel substrates with lamellar puckers skewed the differentiation of MSCs into osteogenic lineages, while the hydrogels with hexagonal patterns were beneficial for adipogenic differentiation [37]. An elegant study demonstrated that ESCs were exquisitely sensitive to nano-topography [38]. The authors observed that nano-topography could exert a significant influence on different behaviors of human ESCs, such as attachment, appearance, propagation, and stemness preservation [38]. Specifically, nano-slick surface cultivation was observed to facilitate pluripotency preservation of ES cells, while nano-rough surface cultivation resulted in spontaneous differentiation of ES cells [38]. Robust propagation platforms for stem cells are expected to allow massive propagation with minimal lot-to-lot variation or be compatible with disinfection procedures [19]. Recent studies have demonstrated the high-efficient expansion of stem cells using microcarriers/stirred suspension bioreactors [39, 40]. Our group recently reported a cell reprogramming-inspired responsive scaffold based on metabolic rewiring and concomitant extracellular pH acidification during the induction of iPSCs, thereby enabling the efficient generation of iPSCs from different types of somatic cells [41]. Future expansion platforms will incorporate state-of-the-art synthetic substrates into advanced microcarriers/bioreactors to realize the potential of massive and low-cost propagation of stem cells.

## Directed differentiation

Lineage specialization of different kinds of stem cells into functional cells of interest lay the foundations for stem cell treatment (Fig. 2B). Effective differentiation into high-purity, terminally differentiated cell types is required to minimize the potentially detrimental influence of undifferentiated progenitor cells or partially specialized cells, which may contaminate terminally mature cells when performed engraftment assay [3]. Just as the mechanical features of substrate materials can be used to facilitate the propagation of stem cells, the mechanical features of substrate materials opt to be adjusted to modulate the targeted specialization of different kinds of stem cells into desired cell types [3].

A ground-breaking study examining malignant transformation in breast cancer demonstrated that biophysical features of substrate materials can elicit a series of effects on cell state [42]. Specifically, breast cancer cells in 3D cultures reverted into a benign morphology after cutting off given integrins, which are plasma membrane-localized proteins that couple the internal force generation mechanisms of cytoskeletal apparatus and ECM [42]. Another seminal study demonstrated that the elasticity of the substrate caused the specialization of MSCs into different lineages [43]. MSCs could differentiate into adipogenic, osteogenic, or myogenic lineages *in vitro* by cultivating matrices with elastic properties being analogs to adipose, bone, or muscle tissue [43]. In addition, Lee and colleagues designed and prepared micropatterned hydrogels targeting integrin interplay to facilitate the attachment and differentiation of MSCs [44]. Beyond materials with static properties, time-dependent mechanical properties of viscoelastic biomaterials have also been demonstrated to function as potent regulators of MSC differentiation [45]. In an elegant study, Chaudhuri and co-workers specifically utilized stress-relaxing biomaterials to show matrix stress-relaxation’s influence on MSC differentiation outcome [46].

The mechanical feature of the substrate also exerts an influence on the differentiation of other stem cells [47, 48] (Fig. 3A). Saha and colleagues studied the influence of biochemical signal, and material modulus on the behaviour of adult NSCs by designing and fabricating synthesized, boundary hydrogel system [47]. Recently, Musah and co-workers described the robust specialization of ESCs into neurons by adjusting the mechanical features of the supporting matrix [48]. To investigate the specialization of human ESCs on scaffolds with different elasticities, the authors designed polyacrylamide hydrogels (0.7, 3, or 10 kPa) and modified them with glycosaminoglycan-binding peptides, thereby facilitating the desirable adhesion of human ESCs [48]. The rigid hydrogels were found to facilitate pluripotency preservation of human ESCs, whereas softer hydrogels led to the reliable specialization of human ESCs into neuronal lineage [48].

In addition to the mechanical parameter of the matrix, other parameters have been demonstrated to modulate the differentiation outcomes of stem cells [49–51] (Fig. 3A). In a typical example, the generation of cellular traction forces is mediated by substrate degradation of encapsulated MSCs, which was required for specialization of MSCs towards osteogenic lineages [49]. Bioactive factors provided by substrate also have a significant influence on the specialization of stem cells [50, 51] (Fig. 3A). In a recent study, Kiskinis and colleagues developed scaffolds based on peptide amphiphile (PA) supramolecular nanofibers displaying biologically active laminin-derived IKVAV peptide, thereby enhancing the generation of matured neurons from stem cells [50]. The authors found that the fabricated substrates caused enhanced activation of β1-integrin-mediated signaling, reduced clustering, and increased arborization, ultimately enabling neuronal maturation with electrophysiological activity [50]. Nie and colleagues reported a biological process-inspired, robust platform to realize the specialization of ESCs into endothelial lineage using steerable chitosan (CS) nitric oxide (NO)-releasing hydrogel [51]. By combining the NO donor in the β-galactose cage and CS backbone, the prepared NO-controlled releasing CS hydrogel was tested in the endothelial fate specialization of ESCs [51]. Of note, the expression of vascular endothelial-type cadherin was upregulated within the tunable NO-releasing condition [51]. Collectively, this study demonstrated that functionalized modifications of biomaterial with functional NO donors represent an effective, uncomplicated approach to specializing ESCs in endothelial lineage [51].

The topography property of bioengineered materials can have significant influences on different aspects of stem cell behaviors [52–54], including attachment, polarization, and propagation (Fig. 3A). Due to the rapid developments of fabrication technologies, substrate topography-induced specialization of stem cells has been a focal point of recent research [52–54]. For example, Lapointe and colleagues investigated the stemness preservation and early specialization of ESCs with thin films comprising gold nanoparticles with a hemispheric topography through fabricating with a blend of alkanethiols [52]. Compared with the behaviour of ESCs cultivated on self-assembled monolayers of the same alkanethiols on vapour-deposited gold, the authors found that nanoscale chemistry and topography affected the subcellular localization of pluripotency-associated factor Oct4 and the expression status of *Fgf5* and *Foxa2* [52]. Follow-up studies revealed endoplasmic reticulum stress and activation of CAV1-YAP signaling as the reason underlying the effects of topographic cues on the specialization of stem cells into osteogenic lineage [53, 54].

## Boosting stem cell engraftment

With the exceptions of the gut [55], cornea [56], skin [57], and liver [58], most adult tissues have a limited ability to regenerate after degeneration or injury [17, 59]. Regenerative therapies involving stem cell transplantation have the potential to apply regenerative treatments to rehabilitate, substitute for, and heal diseased, injured, or aged tissues [17, 59]. So far, stem cell-based treatments have shown modest success clinically except for HSC transplantation [60]. Problems such as undesired cell survival, low cell retention, inefficient differentiation, or inadequate cell implantation into recipient-injured sites pose major challenges [17, 61]. Stem cell behavior is influenced by biochemical or biophysical features of bioengineered materials as discussed earlier in this review. Therefore, the utilization of advanced bioengineered materials provides a way to tackle pre-/post-engrafting issues of cell survival, movement, and engraftment (Figs. 2C and 3B). Here, different kinds of methods on the basis of engineered biomaterials to facilitate stem cell engraftment will be presented. Because of length limitations, we will not list all biomaterials previously reported, but demonstrate important biological principles of different biomaterial-based strategies to enhance stem cell engraftment, highlighting key achievements. In the following sections, we present different approaches that ameliorate survival, specialization of stem cells into desired cell types, influence angiogenesis, and modulate recipient immune responses, with the ultimate goal of improving regeneration outcomes.

### Improving the survival of engrafted donor cells

Surviving of engrafted stem cells at transplantation sites is a prerequisite for the desired outcomes of stem cell regenerative treatments [62]. In general, overall viability for most engrafted donor cells is still low for hours or days when engrafted into recipient sites [62], which greatly limits the therapeutic effect of transplanted cells. Three main mechanisms that cause cell death during and after engraftment have been identified: (i) mechanical stress applied to donors during engraftment, (ii) anchor-dependent death of donor cells and growth factor deficiency, and (iii) inadequate assist from diseased sites of the recipient, such as restricted availability to vascular networks [17]. Engineered biomaterials-based cell delivery systems are amenable to be used to tackle these problems, thereby enhancing the survival of engrafted stem cells [17].

Mechanical stress, including shear and tensile force, is usually neglected but poses great challenges for Newtonian fluid-based delivery methods [63–66], such as phosphate-buffered saline. Since the relatively smaller diameter of the needle, cells experience a greater flow resistance near the wall of a syringe tube, a higher velocity at the center of the syringe, and greater tensile force at the syringe/needle interface [17]. This increase in tensile force is detrimental to the engrafted donors, followed by plasma membrane rupture and elicitation of apoptotic processes, ultimately leading to reduced cell survival immediately after the transplantation process [63–65]. These mechanical stress-mediated effects are evident in terminally differentiated cell types such as neurons [67]. Shear-thinning materials, such as alginate [64], hyaluronic acid [68], and methylcellulose [69] have typical plug flow, preventing mechanical stresses during injection and facilitating the viability of engrafted donors after the injection process. In a typical example, Wang and colleagues developed an injectable scaffold through a dynamic covalent cross-linked method with a creative conjugation of a thermally sensitive, hydrazine-modified elastin-like protein, enabling enhanced mechanical protection to cells encapsulated and quickly restored to reserve engrafted samples homogeneously after injection [70].

Conventional stem cell delivery in buffered saline is often associated with cellular reflux, which is related to interstitial pressure [17]. Some injectable biomaterials gelate rapidly after injection, limiting the phenomenon of cellular reflux, thereby increasing the overall viability of engrafted donors being left over at engrafted site after engraftment [17]. In an elegant study, Ballios and colleagues performed an engraftment assay within a bioresorbable hydrogel mixture of hyaluronan and methylcellulose, thereby yielding a higher efficiency of donor cells engrafted into recipient tissues than that in buffered saline [69]. The improved survival of donor neural progenitor cells was achieved in another study using a bioresorbable hydrogel mixture of hyaluronan and methylcellulose, which is minimally invasive, injectable, and biodegradable [71]. In addition, the peptide-decorated, injectable scaffold dramatically improved the viability of oligodendrocyte progenitor cell samples when delivered to injured rat spinal cords [72]. Human MSCs have been engrafted within an injectable alginate scaffold, rendering approximately 60% retention of initial engrafted samples 24 h post-engraftment into the rat heart, which is in contrast to only 10% using buffered saline [73]. In a recent study, Lee and colleagues reported a collagen dendrimer substrate matrix, modified with diversified pro-surviving bioactive factors via a crosslinked method, thereby enabling length stepwise discharging of the pro-surviving bioactive peptides into the localized transplanted sites to significantly enhance the survival of transplanted cells and functionally improve mouse models of hind-limb ischemia and myocardial infarction [74]. More recently, Martin and colleagues fabricated reactive oxygen species (ROS)-sensitive, polyethylene glycol (PEG)-based hydrogels, cleaning up free radicals to minimize the exposure of human MSCs to cytotoxic ROS as well as to promote length discharging of the loaded cells, which enhances the viability of engrafted MSCs for more than 11 days in contrast to PEG only scaffold or gold-standard, enzymatically sensitive scaffolds [75].

### Preventing anchorage-dependent cell death

With the exception of hematopoietic cells, stem cells must attach to a matrix in order to survive [17]. The pro-survival signaling of attachment is transduced through integrins, a family of plasma membrane-localized attachment receptors, which bind with different constituents of ECM [17]. ECM binding elicits the aggregation of integrins, sensitizes focal adhesion kinase (FAK), and activates the phosphoinositol 3-kinase (PI3K)-protein kinase B (PKB) and downstream MAPK pathways to facilitate the survival of engrafted donor cells [76]. Bioengineered materials functionalized with different ECM molecules that act as ligands for integrin, including fibronectin or hyaluronic acid, can inhibit anchorage-dependent cell death [17]. For instance, cell transplantation in a bioresorbable hydrogel mixture of hyaluronan and methylcellulose significantly improved the survival of transplanted NSPCs or RSCs within recipient sites, which is in contrast to engraftment in buffered saline [69, 71]. The effects of hyaluronan and methylcellulose are transduced in part through hyaluronan recognition with plasma membrane-localized receptor and subsequent sensitization of pro-survival signals [63]. In another example, NSCs transplanted within injectable scaffolds containing either laminin or fibronectin showed improved survival in the traumatic cerebral injury model, which is in contrast to transplantation in media alone [77]. In addition, functional recovery is better in animals transplanted with NSCs encapsulated within scaffolds containing either laminin or fibronectin [77].

Synthetic scaffolds can also be functionalized with ECM-derived peptides with signaling domains that are able to bind to cell surface receptors and mimic ECM-mediated signaling [17]. A typical example is the fibronectin-derived, arginine-glycine-aspartic acid (RGD) peptide, mimicking diversified ECM constituents, such as fibronectin or vitronectin [78, 79]. One practical advantage of using ECM-simulating synthetic molecules, rather than entire forms of ECM proteins, for biomaterial functionalization is that they are amenable to being synthesized by accurate manipulation over composition [80]. On the whole, ECM-simulating synthetic molecules are superior in terms of stability compared with their corresponding entire form [80]. In addition, ECM-simulating synthetic molecule is amenable to accurate spatial or conformational modification of biomaterials via multiple 3D presentation or conjugation strategies [81]. When encapsulated within RGD-functionalized alginate hydrogels, MSC showed better surviving and pro-angiogenic molecular production and *in vivo* ossification in contrast to adhesion-deficient alginate hydrogels [82]. Similar to fibronectin-simulating RGD synthetic molecule, collagen-mimetic peptide, GFOGER [83], and laminin-mimetic peptides, IKVAV [84] or YIGSR [85], have also been used for biomaterial surface functionalization to enhance post-transplantation cell attachment, survival, and function. To achieve maximized cell survival and functionality, ECM-derived peptides need to be precisely formulated for the specific cell population, matching the cell attachment receptors on the plasma membrane [17]. The collagen-simulating synthetic molecule, as an example, has been employed for biomaterial surface functionalization in musculoskeletal tissue [80], and the laminin-simulating synthetic molecule has been utilized for neural tissue [86]. Moreover, the conformation of ECM-simulating synthetic molecules is crucial for their recognition by plasma membrane-localized receptors [80]. On this account, synthetic molecules with larger lengths, cyclic peptides, and/or peptide pairs are usually immobilized on biomaterial surfaces to maximize cellular interaction [80].

In addition to anchorage-dependent signaling, growth factor-dependent signaling is also critical to the survival of engrafted stem cell samples [17]. Pro-survival, anchorage-dependent signaling is transduced through the conjunction of plasma membrane-localized receptors to ECM or ECM-simulating synthetic peptides, thereby activating the FAK, PI3K/PKB, and MAPK pathways [76]. In the case of tissue regeneration, the consignment of growth factors to engrafted stem cells can also contribute to the functional recovery of damaged tissues [17]. Most growth factor ligands are recognized by plasma membrane-localized receptors, which activate downstream PI3K/PKB-MAPK signaling and the subsequent expression of genes involving surviving, propagation, or cell differentiation [87]. Exogenous growth factors rapidly degrade in a culture medium and the conjugation of growth factors to biomaterials by means of appropriate conjugation chemistry can extend the stability and impact of these signalling molecules in stem cell implants [17]. Thus, biomaterials, not only as carriers for stem cells but also as scaffolds for loading and controlling growth factor discharging, are suitable candidates for extending growth factor signaling to maintain high concentrations and prolonged exposure of the transplanted stem cells to growth factors [17].

The desired timing for growth factor discharging depends on an equilibrium between maximizing the viability of engrafted cell samples and easing latent undesired outcomes elicited by prolonged exposure [17]. To give a typical example, bone morphogenetic protein 2 (BMP2), the only ossification-inductive growth factor currently approved by the Food and Drug Administration (FDA), has been widely used for the healing of bone tissue [88]. A retrospective study to determine the rates of BMP2 usage among patients undergoing spinal fusion treatment found that prolonged exposure to supra-physiological doses of BMP2 is relevant to a series of undesired outcomes, including heterotopic ossification, osteoclast-mediated bone resorption, inappropriate angiogenesis, and cervical spine swelling [89]. Excessive dosing and the rapid release of BMP2 are considered to be the main causes of these effects [89]. The releasing kinetics of growth factors has been modulated by adjusting the fixation strategies to biomaterials, of which a synthetic, two-dimensional nano clay represents a typical example [90]. Engineered scaffolds decorated with laponite-sequestered BMP2 were fabricated to significantly lower the efficacious dosage required to induce osteogenesis [91]. Moreover, a hybrid scaffold conjugated with vascular endothelial growth factor (VEGF) and fibroblast growth factor 9 (FGF9) was developed for the consignment of BMP2-producing MSCs *in vivo*, leading to effective neovascularization and bone formation in bone repair [92]. Specifically, the two signaling molecules were fused with fibrin-conjugation peptide, NQEQVSP, thereby allowing covalent recognition by fibrin, which realizes a continual release of VEGF and FGF9, being beneficial to enhanced neovascularization during bone formation [92]. A more recent report described localized consignment of epidermal growth factor (EGF) and basic fibroblast growth factor (bFGF) through dextrin-conjugated growth factors that enable greater and prolonged survival, propagation, or specialization of NSCs through a persistent, easily manipulated discharging of EGF and bFGF [93]. In general, the physical mixing of growth factors with biomaterials often results in an undesirably rapid release, which can be throttled by utilizing chemical conjugation methods to immobilize growth factors, making their release dependent on modifiable dissociation constants [17]. Thus, by adjusting the fixation strategy of physically mixed- or chemically immobilized-growth factors within biomaterials, the discharging kinetics for given growth factors can be adjusted to the varying requirements of tissue regeneration [17].

### Biomaterials-based strategies to create an immune-privileged environment

Various categories of plasma molecules, including albumin, fibronectin, or vitronectin, are deposited on the engrafted surfaces immediately after implantation [94]. These adsorbed molecules play significant roles in the recruitment, attachment, or activation of immune cells at the engrafted site, followed by adverse immune reactions, foreign body responses, and fibrosis, all of which are detrimental to tissue regeneration [94, 95]. The inflammatory immune responses to stem cell implants are regulated by different types of immune cells [95]. Based on their roles in host immune responses, immune cells can be broadly classified into two types: pro-inflammatory types (e.g., T helper 1 [Th1] cells and M1 macrophages) and anti-inflammatory types (T helper 2 [Th2] cells and M2 macrophages) [94, 95]. The status of inflammation and the outcomes of tissue healing is dictated by the equilibrium between different categories of immune cells, with Th1 and M1 macrophages being relevant to pro-inflammatory responses and tissue damage, Th2 and M2 macrophages favoring anti-inflammatory responses, implant-host integration, or tissue regeneration [95]. Bioengineered materials with the ability to regulate macrophage polarization and immunomodulation are promising candidates for tissue healing [95]. For example, Sadtler and colleagues studied how bioengineered material substrates remodel the immune niche in traumatic muscle wounds to facilitate tissue regeneration [96]. To this end, they fabricated tissue ECM substrates to induce regenerative-inducing immune response through mTOR/Rictor-dependent Th2 signalling that guided interleukin-4 (IL-4)-dependent macrophage polarization, ultimately enabling functional muscular repair [96]. More recently, an injectable hydrogel matrix encapsulating modified mesoporous silica nanoparticles for delivering microRNA-21-5p was developed [97]. The authors found that mesoporous silica nanoparticles dramatically attenuated inflammatory reaction by impeding M1 macrophage polarization within the recipient infarcted myocardium, thereby effectively reducing infarct size [97].

**Figure 4.**
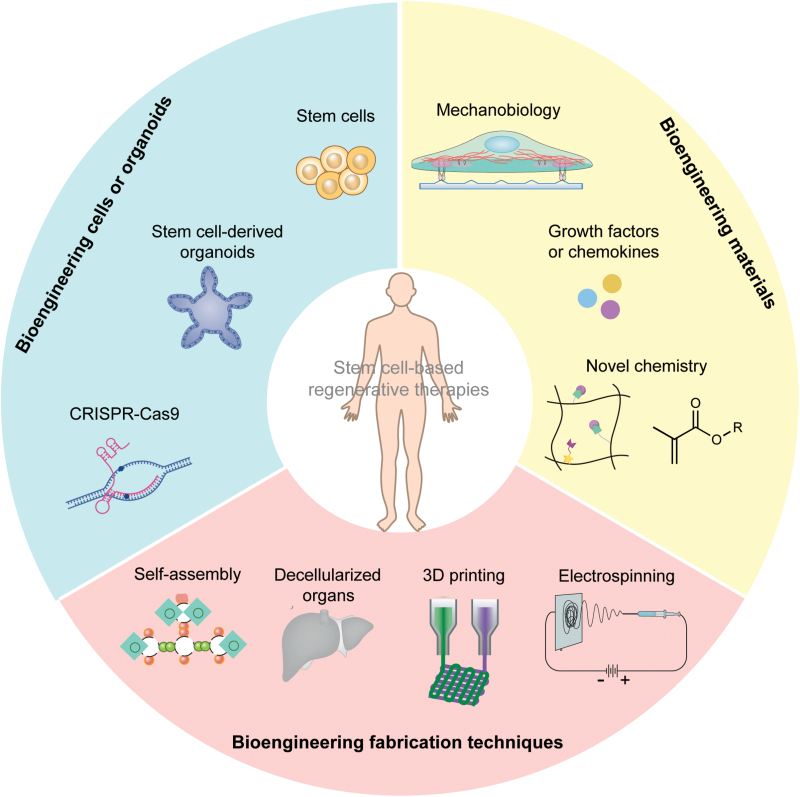
Progress of biomaterial-assisted, stem cell therapies. Stem cells or organoid sources have become available, including pluripotent stem cells, somatic stem cells (such as HSCs, NSCs, and MSCs), and stem cell-derived organoids. Genomic editing tools (such as CRISPR-Cas9) that enable desired cell engineering have also emerged. Advances in understanding the influence of biophysical cues from biomaterials on cellular behavior, innovative growth factor delivery approaches, and novel chemistries, as well as various bioengineering fabrication techniques, boost the progress of stem cell therapies.

Biomaterials can also be used to protect engrafted stem cells from inflammatory responses elicited by the host as immunoisolation apparatus, thereby facilitating the immediate and final outcomes of engrafted allogeneic stem cell samples [95]. To elaborate, the mechanical or biochemical features (including surface topography, matrix stiffness, viscoelasticity, surface coating, porosity, chemical composition, and loaded cytokines) of biomaterials have been flexibly adjusted to elicit favorable immune reactions (Fig. 4A), thereby creating a pro-regenerative environment [98]. Polylactic acid substrate matrices shipped with neurotrophins, such as neurotrophin 3 or brain-derived neurotrophic factor, which are known to orchestrate inflammatory response, have been utilized triumphantly to enhance axon regeneration and axon myelination in spinal cord injury [99]. Another representative example is the treatment of diabetic mellitus by cadaveric islet or insulin-producing β cells produced from PSCs [100]. Conventional transplantation with cadaveric islets has achieved clinical success to some extent, this approach, however, has been hampered by a by-product of immunosuppressive treatment in recipient patients for a long time [100]. Encapsulation of cadaveric islet or PSC-derived β cells into biocompatible materials provides an ideal strategy to address the immunosuppression concern by protecting the transplanted cadaveric donors or β cells produced from PSCs against immune attacks while allowing sustained insulin secretion and glycaemic control [3, 100]. An elegant study has demonstrated glycaemic control in a diabetic model for a long time [100]. PSC-derived β cells were encapsulated within triazole–thiomorpholine dioxide alginate microcapsules capable of mitigating foreign-body responses against the transplanted PSC-derived β cells [100]. In a similar study, the controlled release of human umbilical cord MSC-derived exosomes was realized using hybrid alginate microcapsules [101]. Specifically, exosomes released from the alginate microcapsules into the microenvironment mitigated undesired immune responses, in which xenoengraftment of donor islets loaded within alginate microcapsules resulted in glycaemic control for a long time in the diabetic model [101]. Mechanistic studies have demonstrated that exosomes probably exert immunosuppressive effects by messing up multiple immune-related signaling pathways (such as NF-κB) in myeloid lineages [101].

### Biomaterials-based *in situ* tissue regeneration

Successful clinical therapies involving conventional tissue engineering approaches face several challenges, including immune-compatible, host-acceptable cell sources, scale-up capacity, cost-effectiveness, and preservation/storage issues [102]. In the past 20 years, *in situ* tissue engineering leveraging the body’s endogenous stem cells to revitalize injured tissue has emerged as a new approach [102, 103]. Combined utilization of the revitalized capacities of the body with bioengineered materials is known as biomaterial-based *in situ* tissue regeneration [102, 103]. The purpose of biomaterial design for endogenous tissue regeneration is to coin artificial scaffold platforms mimicking natural tissue, thereby recruiting native stem cells committed to tissue revitalization without eliciting deleterious inflammatory responses, which is undoubtedly a key step towards introducing a safer and more tractable regenerative medicine [102, 103]. The engineered substrate matrix for *in situ* tissue revitalization includes nanoparticulate, hydrogel, monolithic, fibrous, or 3D-printed scaffolds [102]. The engineered substrate matrices interact with tissue at the implanted site through biochemical and biophysical means [102, 103]. The scaffolds’ biochemical cues include the controlled release of chemotactic factors and ions, as well as degradation products of the scaffolds, which can induce specific cellular responses, such as the recruitment and migration of endogenous stem cells to damaged sites and specialization of stem cells into functional cell lineages [102, 103]. In a recent study, the enlistment of endogenous cells to bone defects was successfully triggered through the controlled discharging of stromal cell-derived factor 1α (SDF-1α) [104]. To elaborate, an engineered alginate substrate matrix with defined stiffness customized for bone tissue engineering loaded with SDF-1α, is capable of sustained discharging of SDF-1α, thereby leading to the enlistment of native MSCs and bone revitalization [104]. Xu and co-workers have successfully established chemoattractant gradients within the biomaterials [105]. In this case, the authors induced deep penetration of NSPCs into the matrix through the embedding of semaphorin 3A gradient within Matrigel-based apparatus, promoting the differentiation and maturation of NSPCs and the revitalization of injured sites from cerebral injury [105]. In another example, the controlled discharging of calcium has been achieved with β-calcium silicate/poly-D,L-lactide-glycolide compound substrate matrix, which can activate AMP-activated protein kinase-extracellular signal-related kinases 1/2-RUNX2 signaling axis to promote bone regeneration *in vivo* [106]. Thus, by establishing a proper gradient of chemoattractants or signaling molecules with engineered material-based approaches, endogenous stem cells have been guided to move toward an engrafted site, thereby achieving effective tissue revitalization.

The biophysical characteristics of implanted scaffolds, such as porosity, topological features, micro/nanopatterns, stiffness, viscoelasticity, and degradation kinetics (Fig. 3A) can also influence cell infiltration, cell fate, and regenerative outcome [102]. For example, discrete HA-based micro rods that provide local mechanical cues for cell reprogramming and mitigate cardiac fibrosis after myocardial infarction have been developed [107]. Moreover, the efficient sequestering and controlled releasing of BMP-2 was realized by the functionalization of HA hydrogel with covalently linked bisphosphonate ligands, resulting in effective *in situ* bone regeneration [108]. In a noteworthy study, Darnell and colleagues utilized stress-relaxing biomaterials to show matrix stress-relaxation’s influence on the transplantation outcome of MSCs [109]. Specifically, recipient animals that experienced fast-relaxing hydrogels exhibited superior new bone growth compared with slow-relaxing, stiffness-matched hydrogels [109].

### Bioengineered materials-assisted, *in vivo* cellular reprogramming-based tissue regeneration

Cell fate is changeable and plastic, which was clearly apparent after Yamanaka and colleagues employed a group of transcription factors that determine cell fate and deeply reprogrammed somatic cells into iPSCs [6, 7]. Subsequently, direct cellular reprogramming technology emerged. Different groups of fate-determining transcription factors have been employed to directly transform cell states without first dedifferentiating cells to pluripotent states, thus avoiding the potential for teratoma formation [110]. In injured tissues with limited revitalization ability due to the existence of a large number of terminally differentiated cells or the lack of propagating cells, direct *in vivo* cellular reprogramming to revitalize cells in injured tissues orthotopically to repair tissues is a promising method that does not involve exogenous cell engraftment [102]. Five different methods have been reported for *in vivo* cellular programming: (i) overexpression of fate-determining candidate factors, (ii) gene silencing by RNA interference, (iii) mRNA-mediated cellular reprogramming, (iv) genome editing using clustered regularly interspaced short palindromic repeats (CRISPR) and CRISPR-associated proteins, and (v) epigenetic reprogramming through biochemical and mechanical signals from bioengineered scaffolds [102]. Detailed reviews of these approaches have been provided elsewhere [102, 103]. In this section, we highlight the advancements in direct cellular reprogramming, in addition to the present boundedness and approaching orientations of bioengineered materials-based delivery of lineage-restricted regulatory factors, mRNAs, CRISPR-associated proteins, and epigenetic modifiers for direct cellular reprogramming to elicit *in situ* tissue regeneration.

Since the emergence of direct cellular reprogramming technology, scientists have been committed to understanding the mechanisms of the cellular reprogramming process, so as to improve the efficiency and quality of direct cellular reprogramming [111–114]. A recent study reported the consignment of candidate factors through an adenoviral carrier, demonstrating improved repair of damaged sites in the form of axonal regrowth [115]. Specifically, the authors employed a dual adeno-associated viral system to deliver a group of candidate factors to avoid the potential occurrence of teratomas [115]. Defined cells in the aged retina have been observed to be capable of revitalizing axons in a crush injury model or an experimentally induced glaucoma model [115]. Interestingly, the regenerative effects were associated with active DNA demethylation in defined cells, and inhibition of DNA methylation eliminated the effects of candidate factors on revitalization potential in injured cells [115].

A major obstacle to direct cellular reprogramming is delivery [110]. With the optimization of cellular reprogramming approaches, the current bottleneck is an effective consignment of cell fate-determining candidate factors or mRNAs to defined cell types in targeted tissue [102, 103]. Cell reprogramming entails high-efficient expression of cell fate-determining transcription factors, mRNAs, or microRNAs [102, 103]. Moreover, as cellular reprogramming technology advances, the ideal mixtures for different tissues may have diversified factors, mRNAs, proteins, or small molecule compounds; bioengineering strategies thus will be needed to effectively deliver reprogramming cocktails [102]. Injectable hydrogel substrates, electrospun matrices, microbeads, or synthetic nanoparticles were utilized to realize targeted cellular delivery [102, 103, 116]. Synthetic nanomaterials, as promising candidates, are expected to provide suitable cocktails for endogenous cellular reprogramming, since these formulations are capable of high loading efficiency, stability, biocompatibility, and long shelf life [102, 103]. For instance, degradable nanocapsules were used to deliver myogenic transcription factor (MyoD) into myoblasts to induce myogenic differentiation [117]. The efficiency of myogenic specialization through nanocapsule-mediated consignment was superior to that of the native form of MyoD and is equivalent to the productiveness of MyoD consignment using commercial reagents [117]. An active targeting-based intracellular delivery technique has also been developed to achieve efficient delivery with minimal toxicity [118]. Specifically, Lee and colleagues fabricated DNA-assembled recombinant transcription factors (DART), thereby enhancing the initiative intake of nanoparticles [118]. DART has been found to possess a special signature, which enables it to bind candidate factors, trigger endocytosis in hepatocytes, and elicit endosomal disruption within the acidic environment of the endosome [118]. This study demonstrated the feasibility of the consignment of candidate factors to targeted tissue using nanoparticles [118]. Raftery and colleagues have recently developed a gene-activated substrate matrix that can transmit key signals to endogenous MSCs to prime MSCs for cartilage specialization and inhibition of endochondral ossification, enabling *in situ* articular cartilage defect repair [119]. The intracellular delivery of miRNAs using synthetic nanomaterials for regenerative treatment has also been reported in a noteworthy study [120]. Specifically, miR-222 and neurotrophin-3 were robustly bound into aligned poly(ε-caprolactone-co-ethyl ethylene phosphate)-collagen hybrid substrate matrix, followed by delivery into rats, via cervical incision, to induce axonal regeneration [120].

Unlike other cellular programming approaches that require the entry of reprogramming factors into the nucleus, biomaterial-based mRNA delivery targets the cytosol and is potentially safer considering the low incidence of genomic integration [102, 121]. Mature mRNA that is delivered to the cytoplasmic portion via biomaterials can be readily converted into protein through the ribosome, resulting in elevated expression efficiency, which is suitable for cells that do not divide or slowly divide [102, 103]. In a typical example, the facilitation of ocular restoration by strengthened expression of the targeted protein in experimental animals administrated with lipid nanoparticles loading mRNA compared to control group animals administrated with plasmid DNA has been recently reported [122].

Modulation of endogenous gene expression needed for the transformation of cell fate can be realized via genomic editing using diversified nucleases (e.g., the versatile CRISPR-Cas9 system) [102, 123, 124]. CRISPR-Cas9 mediated genomic editing is an up-and-coming orthotopic tissue revitalization method in terms of easy fabrication, minimal off-target effects, high efficiency, and wide-scale use in different cell types [102]. Besides the commonly used, viral-mediated consignment of CRISPR-Cas9, nanoparticle-mediated consignment methods have recently emerged to facilitate genome editing through CRISPR-Cas9 [102]. In a typical example, a consignment carrier (CRISPR-Gold) composed of gold nanoparticles was developed, thereby enabling direct consignment of target DNA, Cas9 ribonucleoprotein, and PAsp(DET) *in vivo* via local administration in an experimental murine model of Duchenne muscular dystrophy [125]. The authors observed the restoration of expression of dystrophin protein, validating the correction of the dystrophin gene mutation, followed by regenerating functional wildtype dystrophin and improving animal strength [125]. This elegant study underscores the therapeutic potential of gene editing mediated by nanomaterial-based CRISPR-Cas9 for tissue regeneration [125].

The expression status of genes required for the transformation of cell state can also be regulated by epigenetic modifications that alter chromatin structure and the accessibility of DNA without changing the genomic sequence of a target gene [102]. DNA methylation/demethylation and various histone modifications play fundamental roles in configuring chromatin structure to control gene expression [102]. The reversible characteristics of diversified epigenetic modifications render them an up-and-coming method, which may potentially be exploited for orthotopic tissue revitalization [102]. Studies have demonstrated that biophysical cues from bioengineered materials, such as surface topography and stiffness, may alter epigenetic status in target cells, which is followed by cell fate conversion [126–128]. For instance, Downing and colleagues investigated the effects of parallel microchannel mechanical signals on epigenetic states in cells undergoing cell reprogramming [126]. The authors found that cells undergoing reprogramming inoculated on micro-grooved substrate matrices possessed a greater reprogramming potential than cells inoculated on the relatively glossy substrate [126]. This landmark study opens the door to probing the influence of mechanical signals on modulating epigenetic status and subsequent cell fates [126]. In a recent study, trichostatin A (TSA) was incorporated into poly(l-lactic acid)-aligned fiber substrate matrices to promote the structural and mechanical properties of the regenerated Achilles tendon [127]. More recently, TSA was incorporated into a CS-based scaffold, followed by mixing with various proportions (0%, 10%, 20%, and 40%) of biphasic calcium phosphate [128], exhibiting exceptional bone regeneration ability compared with negative controls and commercial bone transplant products [128]. Altogether, those studies demonstrate that the combination of biochemical signals in the form of epigenetic modifier molecules unloaded from engineered substrate matrices with the biophysical clew of engineered substrates can enhance tissue regeneration.

## Improving human cell culture models

The advancements in basic or translational research of biomedicine have been greatly driven by different kinds of model systems [129]. The ideal model system recapitulates the endogenous function and process of the intended natural system, ranging from the microscopic molecular level to the mesoscopic cellular level, and then to the macroscopic organ, or entire organism level [129]. Due to their high throughput, excellent manipulability and low maintenance costs, simplistic models, such as *in vitro* two-dimensional cell cultivation, have been considered as key research means for simulating normal development processes or diversified disorders [129]. Although extensively used in various scenarios of biomedical research, conventional two-dimensional monolayer cell cultures are often assumed to be non-physiological, due to the lacking of physiological crosstalks of inter-cellular or cell-ECM, architectural information, or complexity [23]. In the context of two-dimensional model systems, freshly prepared primary cells will gradually lose some phenotypic and functional features [23, 129]. In recent decades, biomedical research has been mostly conducted in diversified standardized animals, which more closely recapitulates *in vivo* physiology [129]. Although standardized animals enable us to have a more profound grasp of diversified biological phenotypes, there are still disparities in our understanding of the mechanisms of human-specific development, biology, physiology, and disease-related events [130]. In addition, standardized animals are confined by the maneuverability of real-time imaging observations, the presence of mixed variables and relatively limited operability [129]. Moreover, the wide variabilities between humans and the homogeneous genomes of inbred standardized animals create a stark contrast, thereby resulting in insufficient transferability of results from standardized animal-based research to diverse human populations [129, 130].

The advent of 3D culture models is poised to significantly improve the relevance of *in vitro* research. 3D aggregation cultures of MSCs or tumor cells have demonstrated improved utility, despite the lack of organizational information at the tissue level that exists endogenously [131, 132]. Tissue explants or slices may temporarily preserve the cellular architecture and crosstalks, but these model systems are often void of the phenotype in no time [133]. Other 3D cultivation platforms, including cellular spheres, typically lack the ability to support stemness preservation or specialization of stem cells, making it difficult to sustain a 3D culture for extended periods of time [129, 130]. The latest progress in stem cell biology and 3D cultivation techniques have made it possible to derive organoids from stem cells that recapitulate diversified features of endogenous organs [134–136]. Compared with traditional cultivation models or standardized experimental animals, organoids are garnering much attention because of their distinctive self-organizing characteristics from diversified stem cells and their high similarities to endogenous organ counterparts [134–136]. The organoid cultivation platform makes it possible to faithfully dissect normal development processes and multiple disorders, as well as in the identification of new therapeutics [135–137]. The utilization of organoids in developmental biology, disease modelling, or drug screening has been extensively summarized and discussed in several excellent reviews [135–141]. Here, the authors summarize the widespread utilization of organoids generated from stem cells in regenerative medicine.

The development of rudimentary organoids dated from the 1970s, when James Rheinwald and Howard Green successfully generated a stratified, self-organized squamous epithelium by co-culture of newly prepared keratinocytes with proliferation-defective 3T3 feeders [142]. The authors found that cell propagation was limited to the basal layer of the expanding stratified epithelial colonies, while the upper layers were composed of differentiated, keratinized cells that evolved into keratinized envelopes [142]. The method used by this seminal work was simplified by inoculating freshly prepared keratinocytes with irradiated, proliferation-defective feeders under defined cultivation circumstances [142]. The striking progress in the field of ECM and 3D cultivation platforms has laid the foundation for breakthroughs in the field of organoids [134–136]. By 2009, breakthroughs had been made in the field of organoids, with Hans Clevers and colleagues reporting Lgr5(+) ISCs present in the adult gut can propagate, differentiate and undergo the process of self-organization *in vitro* [134]. The authors further designed and fabricated a 3D cultivation platform to reconstruct a niche-like milieu for Lgr5(+) ISCs, so as to obtain murine intestinal organoids [134]. These intestinal organoids can maintain crypt-villus structure and expand for more than 3 months with genomic stability, promoting the isolation and propagation of a large number of organotypic cells [134]. This represents a landmark achievement in the field of organoids, which inspired the subsequent generation of organoids resembling several other organs from either stem cells or primary tissues [143–149].

Organoids have widespread applications in developmental research, disease modeling, tissue engineering, and regenerative treatment [135, 136] (Fig. 4). The first successful attempt to therapeutically reconstitute 3D tissue structure from cultured human stem cells dates back to 1980 when Green and colleagues reported a curative approach utilizing cultured autologous keratinocyte sheets, which were applied to two third-degree burn patients [136]. In the summer of 1983, the therapeutic potential of the method had been demonstrated, which saved the lives of a pair of brothers, who suffered over 95% of their body surface burns [150].

Since the seminal report of intestinal organoids, Yui and co-workers found that murine Lgr5(+) colonic stem cell-derived organoids could be propagated or transplanted into the superficially injured colon with functional recovery at 4 weeks after transplantation [151]. The functional assay was performed in another study using enterospheres derived from murine fetal progenitor cells [152]. Intestinal organoids have also been engrafted into the renal capsule of recipient animals, displaying crypt-villous structure and digestive function, highlighting the potentiality of intestinal organoids for the healing of gastrointestinal disorders, such as short bowel syndrome [153].

In addition to the regenerative potential of intestinal organoids, PSC-derived hepatic organoids can restore hepatic functions as well, thereby improving the survival rate of acute liver failure mice [154]. Exploring the healing and curation of common bile duct diseases by using extrahepatic cholangiocyte organoids to reconstruct extrahepatic bile duct trees has been reported in a noteworthy study [155]. The healed bioengineered bile duct tree was capable of reconstructing native architecture or revitalizing biliary epithelium after engraftment to the renal capsule in recipient immunocompromised mice [155].

Organoids are amenable to being subjected to genome editing technology, serving as a promising method to cure single-gene hereditary disorders [156]. In an elegant study, gene editing of a cystic fibrosis transmembrane conductor receptor (*CFTR*) mutation in ISCs derived from cystic fibrosis patients through the CRISPR/Cas9 gene editing technique was performed [156]. The authors proceeded to demonstrate the functionality of the corrected allele in an organoid system, shedding light on the therapeutic potential of gene correction in patient-derived organoids in other single-gene mutation diseases [156]. Moreover, the use of patient-derived organoids helps to analyze the molecular mechanisms underlying diseases, identify diagnostic phylogenetic markers or develop platforms for individualized drug screening and toxicological evaluations, thereby realizing the prospects of individualized drugs [156]. In another noteworthy research, rectal organoids generated from cystic fibrosis patients have been utilized in preclinical settings to accurately forecast patients’ specific responses to drugs [157].

Although stem cell-derived organoids show exciting promise in applications of regenerative medicine, the current organoid systems exhibit several shortcomings, including a lack of reproducibility for large-scale production, low grade of maturity, or scarce complexity in cellular components when compared with *in vivo* organ counterparts, (partial) deficiency in nerval, vascular, immune, and mesenchymal constituents to recapitulate *in vivo* tissue interactions and inappropriate size of organoids for transplantation assay [138, 158]. The derivation of organoids from PSCs or ASCs mainly relies on the self-organizing properties of PSCs (or ASCs) with limited manipulation over exterior signals applied to organoids [158]. Specifically, the uncontrolled feature of the self-organization process results in significant heterogeneity observed in organoid platforms due to the poor consistencies in hPSC differentiation between different labs [159] and the variability in differentiation outcomes among distinct hPSC lines [160]. The prime target of organoid technology is to improve the morphological and cellular complexity of organoids, provides perfusable vascular networks or facilitate organoid maturation to fully recapitulate the architecture and functionality of endogenous organ counterparts [158]. Bioengineered-controlled microenvironments can realize greater control over organoid self-organization, differentiation, and maturation by integrating the programmability of biochemical signals (bioactive molecules, small chemicals, and ECM-derived attachment molecules) with biophysical cues (stiffness, viscoelasticity, and topography) [158].

During numerous development processes, the relevant morphogens and growth factors are discharged in a spatiotemporal and concentration-dependent manner, which continuously helps to set up polarity and complexity in the structures that present *in vivo* [138]. Similarly, organoids manufactured through continuous local niche simulations facilitate the emergence of polarity and deterministic mode, further driving organoid morphogenesis *in vitro* [158]. Some progress has been made in the reproducible topographic morphogenesis of cerebral organoids [161]. In a seminal study, spatial discharging of Sonic Hedgehog (SHH) has been successfully established through the aggregation of hPSCs and modified hPSCs that inducibly express SHH, allowing for distance-dependent control of SHH concentration and the generation of patterned forebrain organoids [161].

The ultimate realization of organoid maturation and functionality requires the development of bioengineering methods that provide the required nutrition and gas exchange characteristics so that organoids can increase in size, lifespan, maturation, or functionality [158]. In the latest organoid cultivation platforms, nutrition and gas exchange is realized through passive diffusion via culture media, thereby confining organoid size to ~1 mm to prevent the formation of necrotic core [162]. In order to resolve this issue of inadequate nutrition or gas exchange, organoids have been cultivated by researcher scientists within spinning bioreactors [163, 164]. Inducing vascularization within organoids is also becoming another futuristic tactic for addressing organoid nutrition and gas supply issues [162]. An important issue in considering vascularization approaches is how to manufacture endothelial cells for a given organ, followed by being appropriately packaged into blood vessels that sustain organoid functionality [162]. The transcriptional and epigenetic signatures that govern endothelial cell differentiation in the brain, lungs, liver, or kidneys have recently been addressed [165]. With this in mind, researchers have achieved the vascularization of hPSC-derived organoids by introducing endothelial cells [148] or administrating VEGFs [166]. Of note, Wimmer and colleagues have successfully established human vascular organoids from hPSCs, enabling them to assemble into assembloids with other types of organoids, thereby achieving vascularized organoids [167]. Another strategy for guiding vascularization in organoids is implantation, where host blood vessels infiltrate into the implanted organoids [162]. *In vivo* vascularization of hPSC-derived brain organoids [168], kidney organoids [169], or liver buds [170, 171] has been achieved to at least some extent, after engraftment into recipient animals.

Inducing vascularization using microfluidic technology represents an alternative bioengineering approach [162]. In a seminal report, Homan and co-workers fabricated a microfluidic platform to promote the expansion of endothelial progenitor cells and facilitate the induced formation of vascular networks [172]. The authors also demonstrated that these vascularized kidney organoids exhibited morphological and functional maturation [172]. In another noteworthy study, Cho and colleagues successfully ameliorated human brain organoid cultivation by reconstructing cerebral-simulating niches with the well-designed microfluidic platform, enabling the robust cultivation of mature organoids for effective modeling and drug screening for various nervous disorders [173]. The fabrication of convective channels by 3D bioprinting offers another possible method to create vascular networks [162]. A recent report described a robust method to assemble a large number of organ building blocks into tissue matrices, where perfusable interconnected networks are established through well-designed bioprinting [174].

Regardless of the approaches mentioned above, it is essential to achieve capillary systems in organoids when inducing vascularization, i.e., the vascularized networks are expected to remain stable over time and possess a composition and morphology similar to endogenous vasculature [162].

The primary condition of organoid cultivation is to provide essential biochemical signals or biophysical cues while supporting the self-organizing process [158]. Most cultivation platforms for organoids depend on animal-derived ECM, e.g., Matrigel [3, 21, 158]. However, the unclear composition, heterogeneity, and variability between batches of Matrigel impede the practical usage in organoid cultivation [22, 158]. Alternative methods focused on engineered matrices that simulate key properties of ECMs from native tissues [158]. In a typical example, 3D niche microarrays have been developed to evaluate the influence of engineered matrices with diversified stiffness, degradability, or topography on the cultivation of organoids [175]. Synthetic matrices have also been used to replace the highly variable Matrigel to maintain the cultivation of diversified organoids derived from PSCs or ASCs [140, 176–180]. In a typical example, poly(lactide-co-glycolide) copolymer microfilament substrates have been developed to improve cortical development in human brain organoids [140]. More recently, Shao and colleagues generated human amnion-like tissue *in vitro* through the self-organizing of PSCs within a bioengineered niche [176].

Naturally occurring materials-derived matrices [177, 178], synthetic polymer materials [33, 179], and recombinant protein-engineered hydrogels [180] have been fabricated to replace the undefined tumor-derived ECM (Matrigel) to support organoid culture and improve the translational relevance of organoids. Specifically, intestinal organoids derived from iPSCs have been maintained in alginate hydrogel with no inherent cell instructive properties [177], which demonstrates mechanical feature alone is ready to cultivation of intestinal organoids [177]. In addition, a fibrin/laminin composite matrix was fabricated by Broguiere and colleagues to support the propagation of epithelial organoids [178], making the composite matrix a promising candidate for organoid culture as a defined equivalent to Matrigel. Moreover, DiMarco and co-workers developed a scaffold containing elastin-like protein to facilitate the formation of intestinal organoids [180]. The authors found that soft scaffolds with higher concentrations of RGD ligands exhibited enhanced support for organoid cultivation compared with stiff scaffolds with low concentrations of RGD [180].

A typical synthetic polymer material is the PEG-based hydrogel [158, 181]. PEG-based hydrogels are up-and-coming candidates for steady culture for organoids in terms of good biocompatibility, commercial availability, facile chemical functionalization with biological ligands and signaling molecules, and crosslinking capacity, permitting more robust experimentation [181]. For example, García and colleagues developed a chemically defined substrate matrix from a four-armed PEG macromer (PEG-4MAL) capped with maleimide to support the steady propagation of intestinal organoids [179]. This chemically defined substrate matrix was further used to deliver organoids by the authors for *in vivo* functional assay, which achieved effective colonic wound closure [179]. In another seminal study, a PEG-based substrate matrix platform with biophysical characteristics was developed to maintain the formation or propagation of intestinal organoids at different stages [33]. The optimal conditions for the propagation of ISCs or formation of organoids were further investigated using modular, chemically defined scaffolds with adjustable stiffness, degradability, or bioactive molecules [33]. Based on these observations, the authors have set up a chemically defined cultivation platform to efficiently propagate ISCs [33]. Importantly, the general design principles to fabricate a chemically defined scaffold for intestinal organoids presented by this study are amenable to be applied to identify scaffolds, which are endowed with the capability to cultivate other ASCs or stem cell-derived organoids [33].

## Conclusions and outlook

Recent years have witnessed the broad applications of stem cell therapies to treat degenerative diseases, hematoma, and even some intractable diseases, with several exciting clinical applications now on the horizon [3, 12]. A prime example highlights how stem cell-based treatment can profoundly affect clinical outcomes: HSC engraftments have been performed for the treatment of patients with multiple myeloma, lymphoma, leukemia, and autoimmune diseases for over 60 years [13]. This procedure has been facilitated through advancements in matching human leukocyte antigen genes or engrafted approach [13]. In another typical example, the life-saving regeneration of more than 80% of the surface area of the damaged epidermis of a 7-year-old child suffering from a devastating form of junctional epidermolysis bullosa was achieved by means of autologous transgenic epidermal stem cell implantation [14], which supplies classic workflow that may be extended to other forms of stem cell-based treatment. Moreover, Dekkers and co-workers successfully generated cystic fibrosis patient-derived organoids and employed patient-derived organoids to evaluate therapeutic drugs that could reverse disease progression [157].

The significance of bioengineered materials in stem cell therapies is more and more apparent [3] (Fig. 4). The substantial development in biomanufacturing techniques has made it possible to fabricate bioengineered materials with manipulations of on-demand mechanical or biochemical cues [102]. 3D bioprinting can accurately deposit cells, engineered biomaterials, or bioactive molecules into customized scaffolds to model endogenous tissue frameworks compared with traditional techniques [182], thereby eliciting the recruitment or migration of native cells and the spatiotemporal establishment of morphogenic cues, thus accelerating tissue regeneration [182].

The ideal biomaterial used for tissue regeneration is expected to possess a degradation profile that matches the healing procedures of injured sites within a unique niche [183]. Faithfully simulating the healing procedures can be achieved through biomaterial design that adapts to changes in cells undergoing tissue repair and subsequently alters its characteristics based on biochemical signals or biophysical cues [183]. Next-generation, multifunctional biomaterials with state-dependent, dynamically bioresponsive properties can be fabricated utilizing chemistry, architecture, and configuration of scaffold building modular to manufacture biomaterials [183].

Minimally invasive consignment of treatments is an up-and-coming method to facilitate functional restoration of injured sites [102]. The consignment of healing treatments including microneedles or engineered biomaterials has been realized through minimally invasive methods, which have diversified advantages such as mitigated discomfort, retention of healing activities, or durative delivery for effective treatment [102]. For instance, Yang and co-workers described a removable microneedle-mediated consignment platform for sustained and efficient consignment of exosomes and activators of hair follicle stem cells, thereby enhancing treatment effectiveness and hair regrowth [184]. Another approach to transport therapeutics to diseased/injured sites in a minimally invasive manner is through injectable biomaterials [185–189].

Host responses significantly influence the destiny of manufactured materials implanted for tissue regeneration within the body [95]. The engagement of immune cells in tissue repair has been harnessed to improve tissue regeneration outcomes [95]. For example, biofunctionalized scaffolds were employed in a seminal study to sequentially deliver cytokines to promote the transition of macrophages from M1 to M2, thus secreting angiogenic factors with proper doses and timing to facilitate vascularization in engineered building blocks [189]. In recent years, the importance of the immunomodulatory effects of bioengineered materials on macrophage polarization during tissue regeneration has been increasingly recognized [95, 183]. It is expected that innovative bioengineering strategies for manufacturing immunomodulatory materials will be set up, thereby eliciting favorable host responses and achieving successful tissue regeneration.

Non-invasive systems for detecting the behavior of implanted biomaterials within the host, enabling a real-time assessment of cell-biomaterial interactions during tissue regeneration, are also expected to emerge [183]. The interactions among cells, implanted biomaterials, and the surrounding matrix have led to the concept of ‘materiobiology’ [183, 190]. Furthermore, omics-based techniques, including genomics, epigenomics, or proteomics have been used, which accelerate unbiased understanding of the interactions among cells, implanted biomaterials, and the surrounding matrix during tissue regeneration [191]. In addition, we can gain additional insights into the detailed events occurring in cell-biomaterial interactions during tissue regeneration using single-cell sequencing with unparalleled precision and accuracy [192]. Artificial intelligence-based machine learning is also expected to eventually predict the outcomes of biomaterial-assisted, stem cell-based regenerative treatment.

Last but not least, quality control is of critical importance in monitoring the manufacture of diversified stem cells [193, 194]. National or international quality control systems have been established, which guarantee the strict quality of stem cell-based therapeutic products for regenerative medicine [193–199]. Quality control systems include the policies of good manufacturing practice, which refers to different stages of stem cell assembly, handling, deposit, training of personnel, and equipment of the cell processing laboratory [193, 194].

The comparison of iPSC lines from different labs is decided by the consistency of critical quality attributes (CQAs), which include but are not limited to cell characteristics, asepsis, endotoxin, genomic stability, cell expansion-associated epigenetic changes, characterization, and potency [195–199]. Comparison of iPSC lines also requires the agreement of validations of appropriate CQA measurements and the further establishment and constant revisions of standards [200].

Several quality control strategies have already been developed for diversified stem cells. In an elegant study, Baghbaderani and colleagues developed a step-by-step cGMP-compliant process for the manufacturing of clinically compliant iPSC lines [201]. In another study, Skorska and co-workers developed an on-site production program for CD133-positive stem cells to treat ischemic heart diseases [202]. The system of standardized clinical practices has been established to make it possible to yield a large number of MSCs from bone marrow aspirate to treat patients [203]. More recently, Hirose and colleagues achieved cell tracking automatically based on deep learning, enabling quality control of keratinocyte stem cells in a non-invasive manner [204]. People are increasingly attaching importance to quality control systems for diversified stem cells, providing guarantees for stem cell-based treatments [200]. Artificial intelligence or machine learning technologies could further aid the development of quality control systems for stem cells [200].

**Figure 5.**
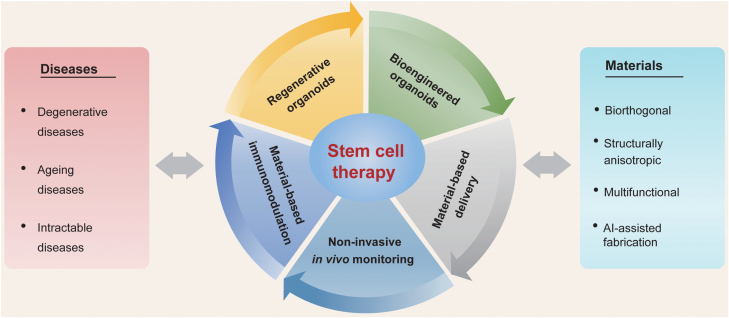
Prospects for biomaterials-assisted, stem cell-based regenerative therapies.

In summary, engineered biomaterial-assisted, stem cell-based regenerative therapy is a burgeoning, interdisciplinary field. Future engineered biomaterials may serve not only as structure support or delivery vehicle for cells or bioactive factors but also as multifunctional regulators of angiogenesis, neurogenesis, and inflammatory responses to maximize the outcome of stem cell treatments (Fig. 5). With diversified biomaterials are fabricated for stem cell-based regenerative treatments, standards, and qualifications are expected to be established to form large-scale, commercial systems. Also, the issues of accessibility and affordability must be considered when translating stem cell-based regenerative therapy to clinical use. Overcoming these challenges will transform engineered biomaterials-assisted, stem cell-based regenerative strategies into therapies that meet the urgent needs for treating degenerative, aging, and other intractable diseases.
